# Diagnostic Performance of the AptoDetect™-Lung Biomarker for Lung Cancer in a High-Risk Korean Population: A Multicenter Prospective Study

**DOI:** 10.3390/biomedicines14071423

**Published:** 2026-06-23

**Authors:** Da Som Jeon, Chang Dong Yeo, Chi Young Kim, Jung Seop Eom, Wonjun Ji, Min Jee Kim, Jung-Min Kim, Seong Hoon Yoon, June Hong Ahn, Jun Hyeok Lim, Chaeuk Chung, Dong Won Park, Seung Hyeun Lee, Chang-Min Choi

**Affiliations:** 1 Division of Pulmonology and Critical Care Medicine, Department of Internal Medicine, Asan Medical Center, University of Ulsan College of Medicine, Seoul 05505, Republic of Korea; mickyhoney@naver.com (D.S.J.); jack1097@naver.com (W.J.); minjee60864@gmail.com (M.J.K.); 2 Division of Pulmonology and Critical Care Medicine, Department of Internal Medicine, Hanyang University Guri Hospital, Hanyang University College of Medicine, Guri 11923, Republic of Korea; 3 Division of Pulmonology, Department of Internal Medicine, Eunpyeong St. Mary’s Hospital, College of Medicine, The Catholic University of Korea, Seoul 03312, Republic of Korea; brainyeo@catholic.ac.kr; 4 Division of Pulmonology, Department of Internal Medicine, Gangnam Severance Hospital, Yonsei University College of Medicine, Seoul 06273, Republic of Korea; cykim@yuhs.ac; 5 Department of Internal Medicine, Pusan National University Hospital, Pusan National University School of Medicine, Busan 49241, Republic of Korea; ejspulm@gmail.com; 6 Aptamer Sciences Inc., Seongnam 13487, Republic of Korea; mserk@aptsci.com; 7 Division of Pulmonology, Allergy, and Critical Care Medicine, Department of Internal Medicine, Pusan National University Yangsan Hospital, Pusan National University School of Medicine, Yangsan 50612, Republic of Korea; drysh79@gmail.com; 8 Division of Pulmonology and Allergy, Department of Internal Medicine, Yeungnam University Medical Center, Yeungnam University College of Medicine, Daegu 42415, Republic of Korea; fireajh@gmail.com; 9 Division of Pulmonology, Department of Internal Medicine, Inha University Hospital, Inha University School of Medicine, Incheon 22332, Republic of Korea; jhl@inha.ac.kr; 10 Division of Pulmonology, Department of Internal Medicine, Chungnam National University Hospital, Chungnam National University College of Medicine, Daejeon 35015, Republic of Korea; universe7903@gmail.com; 11 Division of Pulmonology and Allergy, Department of Internal Medicine, Shihwa Medical Center, Namchon Medical Foundation, Siheung 15034, Republic of Korea; dongwonpark@hanyang.ac.kr; 12 Division of Pulmonary, Allergy, and Critical Care Medicine, Department of Internal Medicine, Kyung Hee University Hospital, College of Medicine, Kyung Hee University, Seoul 02447, Republic of Korea; 13 Division of Oncology, Department of Internal Medicine, Asan Medical Center, University of Ulsan College of Medicine, Seoul 05505, Republic of Korea

**Keywords:** lung nodule, biomarker, lung cancer screening, aptamer

## Abstract

**Background/Objectives**: Blood-based biomarkers may improve risk stratification of indeterminate pulmonary nodules detected on low-dose computed tomography (LDCT). We evaluated the diagnostic performance and independent predictive value of an aptamer-based blood assay, AptoDetect™-Lung, in a high-risk Korean screening population. **Methods**: This multicenter prospective cohort study enrolled adults with Lung Imaging Reporting and Data System (Lung-RADS) category 3 or 4 pulmonary nodules identified on LDCT across ten tertiary hospitals in South Korea between June 2023 and December 2024. Analyses focused on a predefined high-risk subgroup meeting Korean screening criteria (age 54–74 years and ≥30 pack-years of smoking). Baseline serum AptoDetect™-Lung scores were measured. Associations with lung cancer diagnosis were assessed using univariate and multivariable logistic regression, adjusting for clinical and radiologic variables. Diagnostic performance was evaluated using receiver operating characteristic analysis. **Results**: Among 1084 participants with histopathologic confirmation, 319 met high-risk criteria, of whom 260 (81.5%) were diagnosed with lung cancer. In this subgroup, the AptoDetect™-Lung score was independently associated with malignancy after adjustment (adjusted odds ratio of 1.14 per unit; 95% confidence interval of 1.02–1.27; *p* = 0.020). Discriminative performance was higher in the high-risk subgroup than in the overall cohort (area under the curve [AUC] of 0.639 vs. 0.570; *p* = 0.025). Performance was higher for squamous cell carcinoma and small-cell lung cancer than for adenocarcinoma. A multivariable model incorporating biomarker score, Lung-RADS category, age, and family history achieved an AUC of 0.710. **Conclusions**: An aptamer-based blood biomarker may provide modest adjunctive value for risk stratification in high-risk individuals.

## 1. Introduction

Low-dose chest computed tomography (LDCT) is widely implemented for lung cancer screening and has been shown to reduce lung cancer-related mortality, particularly among individuals at high-risk due to age and smoking history [1,2]. However, a substantial proportion of pulmonary nodules detected on LDCT are classified as indeterminate [3], limiting accurate risk stratification based on imaging findings alone [4].

Routine invasive biopsy for all detected nodules is neither feasible nor desirable, given the associated procedural risks, potential complications from unnecessary interventions, and increased healthcare costs. Conversely, reliance on serial imaging surveillance may delay definitive diagnosis [5]. These challenges have driven growing interest in blood-based biomarkers as noninvasive adjuncts to imaging, with the potential to improve early lung cancer detection and refine risk stratification among individuals with indeterminate nodules [6,7]. Existing blood-based approaches, including ctDNA-based liquid biopsy, have been primarily validated in advanced-stage disease and may have limited sensitivity in early-stage or low-shedding tumors commonly detected on screening CT [8,9]. A serum protein-based aptamer assay may provide complementary biological information by capturing tumor-associated and host-response signals, including inflammatory, immune, and tissue-remodeling pathways. Therefore, aptamer-based proteomic profiling should be interpreted as an adjunctive risk-stratification tool rather than a replacement for established imaging or tissue-based diagnostic pathways.

AptoDetect™-Lung is an aptamer-based blood assay designed to assess a panel of protein biomarkers associated with lung cancer biology [10,11]. Prior studies have demonstrated its diagnostic potential, with Jung et al. reporting improved discrimination among screening-eligible high-risk individuals aged ≥55 years with a smoking history of ≥30 pack-years [12]. However, much of the existing evidence is derived from retrospective analyses or studies involving heterogeneous populations with pretest risk [12,13]. As a result, the potential diagnostic value of blood-based biomarkers beyond LDCT has not been fully validated in prospective, real-world screening settings.

In South Korea, the national lung cancer screening program defines high-risk individuals as those aged 54–74 years with a smoking history of at least 30 pack-years [14]. Accordingly, we conducted a multicenter prospective cohort study to evaluate the diagnostic performance of AptoDetect™-Lung in patients with Lung Imaging Reporting and Data System (Lung-RADS) category 3 or 4 pulmonary nodules detected on LDCT. We specifically focused on high-risk individuals and aimed to identify independent predictors of lung cancer using multivariable models incorporating Lung-RADS categories, quantitative imaging features, and relevant clinical variables. Through this approach, we sought to assess the potential adjunctive value of an aptamer-based blood biomarker in routine lung cancer screening practice.

## 2. Materials and Methods

### 2.1. Study Design and Participants

This multicenter, prospective cohort study was conducted across ten tertiary referral hospitals in South Korea between 13 June 2023 and 9 December 2024. Eligible participants were adults (≥19 years) with pulmonary nodules classified as Lung-RADS version 2022 categories 3 or 4 on chest CT, as reviewed by board-certified thoracic radiologists. Exclusion criteria included pure ground-glass nodules, active pulmonary infection, a history of malignancy within the past 5 years (except adequately treated non-melanoma skin cancer or carcinoma in situ), and pregnancy.

**Figure 1:**
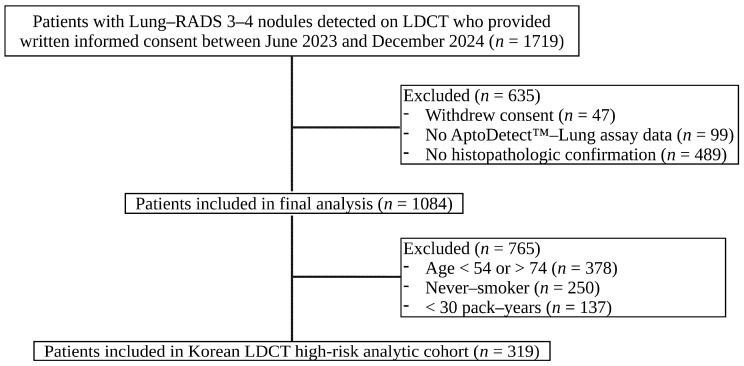
Flow diagram of participant enrollment and high-risk subgroup selection. Of 1719 initially enrolled participants with Lung-RADS 3–4 nodules detected on LDCT, 1084 participants with available AptoDetect™-Lung assay data and histopathologic confirmation were included in the final analytic cohort. The high-risk subgroup (*n* = 319) was defined according to Korean national lung cancer screening criteria: age 54–74 years and smoking history of ≥30 pack-years. LDCT, low-dose computed tomography; Lung-RADS, Lung Imaging Reporting and Data System.

Of 1719 initially enrolled participants, 47 withdrew consent, leaving 1672 individuals for screening. After excluding participants without available AptoDetect™-Lung assay data (*n* = 99) or histopathologic confirmation (*n* = 489), a total of 1084 individuals remained eligible. The requirement for histopathologic confirmation, while necessary for definitive outcome ascertainment, excluded a large proportion of participants with lower-suspicion nodules who were managed with surveillance rather than biopsy. For the present analysis, we further restricted the cohort to a high-risk subgroup defined by the Korean LDCT screening criteria. Although the national guideline also includes smoking cessation within the past 15 years, this criterion was not applied because smoking cessation duration was not consistently documented in the study dataset and therefore could not be reliably ascertained. Ultimately, 319 participants met the high-risk criteria and were included in the final analysis (Figure 1).

### 2.2. AptoDetect™-Lung Assay and Sample Processing

The AptoDetect™-Lung assay (model ALUC-1024; Aptamer Sciences Inc., Seongnam, Republic of Korea) is a multiplexed, aptamer-based bead array platform that quantifies seven serum proteins (C9, CA6, EGFR1, MMP7, SERPINA3, KIT, and CRP) [10,11,15]. Aptamers are chemically modified oligonucleotides with high affinity and specificity for their protein targets, enabling simultaneous multiplexed detection. These proteins were selected based on prior aptamer-based discovery and validation studies demonstrating differential serum expression between patients with lung cancer and controls, and collectively reflect multiple biological processes relevant to lung cancer and tumor–host interactions, including inflammatory, immune, growth factor-related, and tissue-remodeling pathways [10,11,15]. In brief, target-specific aptamers are immobilized and incubated with serum samples to allow aptamer–protein binding. After removal of unbound components, target-bound aptamers are released via photocleavage and hybridized with bead-conjugated capture probes and biotin-conjugated detection probes. Fluorescence signals are then generated and measured as mean fluorescence intensity values using a Luminex-based reader. Individual protein concentrations are converted into numerical scores, and a proprietary algorithm integrates these values to generate a composite risk score ranging from 0 to 10. In accordance with manufacturer specifications, a score ≥ 5 was defined as high risk, whereas a score < 5 was considered low risk.

Sample processing was standardized across all participating institutions to minimize pre-analytical variability. At baseline, 4 mL of peripheral blood was collected in serum-separating tubes for AptoDetect™-Lung testing. Samples were processed within four hours of collection, centrifuged, and aliquoted serum was stored at −70 °C until analysis. To further reduce inter-laboratory variability, all assays were performed centrally at Asan Medical Center (Seoul, Republic of Korea), where assay calibration and quality control procedures were performed according to standardized protocols.

### 2.3. Statistical Analysis

The normality of continuous variables was assessed using the Shapiro–Wilk test. Continuous variables are presented as mean ± standard deviation (SD) or median with interquartile ranges (IQRs), as appropriate. Variables that did not satisfy normality assumptions were compared using the Mann–Whitney U test. Categorical variables are summarized as counts and percentages and were compared using the chi-square or Fisher’s exact test, as appropriate.

Diagnostic performance was assessed using receiver operating characteristic (ROC) curve analysis, with calculation of sensitivity, specificity, positive predictive value (PPV), negative predictive value (NPV), and the area under the curve (AUC) with corresponding 95% confidence intervals (CIs).

Univariable logistic regression analyses were first conducted to identify factors associated with lung cancer. Variables with *p* < 0.10 in univariate analyses were considered for inclusion in the multivariable analysis. Adjusted odds ratios (OR) with 95% CIs were reported, with continuous predictors modeled per 1-unit increase.

Model performance was internally evaluated using bootstrap resampling with 1000 iterations. In each iteration, a bootstrap sample of equal size was drawn with replacement from the original dataset, and the logistic regression model was refitted in the bootstrap sample. Model discrimination was evaluated using the AUC. Calibration was assessed using the Hosmer–Lemeshow goodness-of-fit test, calibration intercept, calibration slope, and Brier score. The Hosmer–Lemeshow test was performed by grouping patients into 10 groups based on deciles of predicted probability and comparing observed and expected malignancy frequencies. The Brier score was calculated as a measure of overall prediction error.

All statistical tests were two-sided, and a *p*-value < 0.05 was considered statistically significant. All statistical analyses were performed in R (version 4.5.1; R Foundation for Statistical Computing, Vienna, Austria). Receiver operating characteristic (ROC) curves were generated using the pROC package (version 1.18.5) [16], and 95% confidence intervals were estimated using the DeLong method. ROC analyses for the total cohort, high-risk subgroup, and histology-specific subgroups were based on the AptoDetect™-Lung score as a continuous predictor. In the histology-specific analyses, each histologic subtype was compared with non-malignant cases within the high-risk subgroup. For the multivariable logistic regression model, ROC analysis was performed using the predicted probabilities derived from the final model.

### 2.4. Ethical Considerations

This study was approved by the Institutional Review Boards of all participating centers (IRB No.: KHUH 2022-10-033-003) and was conducted in accordance with the Declaration of Helsinki. Written informed consent was obtained from all participants before enrollment.

## 3. Results

### 3.1. Study Population and Baseline Characteristics

A total of 1719 participants with LDCT-detected pulmonary nodules were prospectively enrolled across ten tertiary referral centers. After excluding participants with unavailable AptoDetect™-Lung assay data (*n* = 99), without histopathologic confirmation (*n* = 489), or who withdrew consent (*n* = 47), the final analytic cohort comprised 1084 participants. Of these, 868 had malignant disease (80.1%) and 216 had benign lesions (19.9%). A high-risk subgroup was defined according to the Korean national lung cancer screening criteria (age 54–74 years with a smoking history of ≥30 pack-years), comprising 319 participants (29.4% of the total cohort). Within this high-risk subgroup, 260 participants had malignant disease (81.5%) and 59 had benign lesions (18.5%).

Baseline characteristics are summarized in Table 1. Compared with the overall cohort, high-risk individuals were predominantly male (96.9% vs. 68.1%, *p* < 0.001) and had greater cumulative smoking exposure (median 42.0 vs. 37.5 pack-years, *p* < 0.001). Eastern Cooperative Oncology Group (ECOG) performance status, body mass index (BMI), personal history of cancer, familial history of cancer, pulmonary comorbidities, and Lung-RADS category distribution were similar between the two groups.

Median AptoDetect™-Lung scores did not differ significantly between the overall cohort and the high-risk subgroup (4.2 vs. 4.4, *p* = 0.37), nor did the proportion of participants classified as high risk (≥5: 42.2% vs. 43.9%, *p* = 0.63).

Histologic subtype distribution differed significantly between cohorts (*p* < 0.001). Compared with the total cohort, the high-risk subgroup demonstrated higher proportions of squamous cell carcinoma (37.7% vs. 22.4%) and small-cell lung cancer (15.0% vs. 10.0%), with correspondingly lower prevalence of adenocarcinoma (39.6% vs. 61.6%). These patterns reflect the established association between heavy smoking and non-adenocarcinoma lung cancer histologic subtypes.

### 3.2. Univariate and Multivariable Analysis of Malignancy Predictors

Univariate logistic regression analyses identified several variables associated with malignancy within the high-risk subgroup (Table 2). The AptoDetect™-Lung score was significantly associated with malignancy (odds ratio [OR] 1.19 per 1-point unit increase; 95% confidence interval [CI], 1.07–1.32, *p* = 0.001), corresponding to a 19% increase in the odds of malignancy per unit increase in score. High-risk Lung-RADS categories (4B/4X vs. 3/4A: OR 2.91 [95% CI, 1.30–6.50], *p* = 0.009) and older age (OR 1.08 per year [95% CI, 1.03–1.14], *p* = 0.003) were also significantly associated with malignancy. Familial history of cancer showed a borderline association (OR 1.85 [95% CI, 0.97–3.55], *p* = 0.064).

In contrast, personal history of cancer (*p* = 0.518) and ECOG performance status ≥ 2 (*p* = 0.738) were not significantly associated with malignancy. Consistent with standard epidemiologic practice, variables with *p* < 0.10 in univariate analyses were selected for inclusion in the multivariate model to ensure adequate adjustment for potential confounding. In multivariable logistic regression analyses, the AptoDetect™-Lung score, Lung-RADS category, age, and family history of cancer all remained independently associated with malignancy (Table 3).

The AptoDetect™-Lung score retained statistical significance after adjustment for clinical and imaging variables (adjusted OR 1.14 per 1-point increase; 95% CI, 1.02–1.27; *p* = 0.020), confirming its independent predictive value beyond conventional risk factors. High-risk Lung-RADS categories (adjusted OR 2.73; 95% CI, 1.16–6.42; *p* = 0.022) and age (adjusted OR 1.08 per year; 95% CI, 1.03–1.15; *p* = 0.005) also remained significant predictors.

Notably, family history of cancer achieved statistical significance in the adjusted model (adjusted OR 2.17; 95% CI, 1.09–4.31; *p* = 0.027), despite only borderline significance in univariate analysis (*p* = 0.064). This finding suggests that family history of cancer represents an independent risk factor, the association of which becomes more apparent after controlling for confounding variables. The full model demonstrated overall statistical significance (AIC = 288.3, McFadden’s R^2^ = 0.089, likelihood ratio χ^2^ = 27.2; *p* < 0.001). The multivariable model showed an apparent AUC of 0.710. Internal model performance was assessed using bootstrap resampling with 1000 iterations. The Hosmer–Lemeshow goodness-of-fit test showed χ^2^ = 18.81 with 8 degrees of freedom (*p* = 0.016), indicating some departure from perfect calibration. However, the calibration intercept was 0.007, and the calibration slope was 0.991, close to their ideal values of 0 and 1, respectively. The Brier score was 0.139.

### 3.3. Diagnostic Performance and ROC Analysis

**Figure 2:**
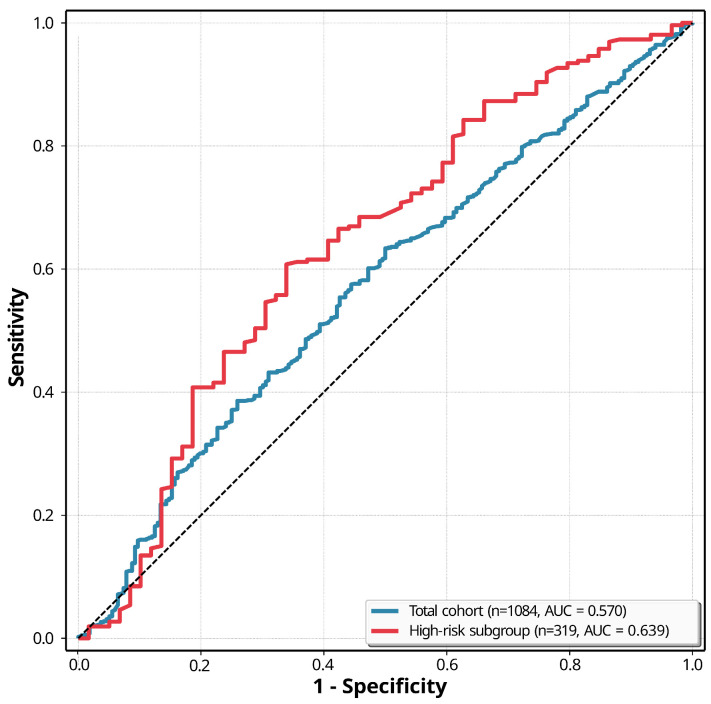
Receiver operating characteristic (ROC) curves of the AptoDetect™-Lung assay in the total cohort and the high-risk subgroup. ROC curves compare the discriminative performance of AptoDetect™-Lung for detecting malignancy in the total cohort (*n* = 1084; AUC 0.570; 95% CI, 0.529–0.612) and the high-risk subgroup (*n* = 319; AUC 0.639; 95% CI, 0.566–0.712; *p* = 0.025 for AUC comparison). The diagonal dotted reference line represents chance-level discrimination (AUC = 0.5). AUC, area under the curve; CI, confidence interval; ROC, receiver operating characteristic.

Receiver operating characteristic (ROC) curve analyses were performed to evaluate the discriminative performance of the AptoDetect™-Lung assay (Figure 2). In the overall cohort, the AUC was 0.570 (95% CI, 0.529–0.612). Discriminative performance was modestly higher in the high-risk subgroup, with an AUC of 0.639 (95% CI, 0.566–0.712, *p* = 0.025 compared with the total cohort), indicating higher discrimination in an enriched high-risk population.

**Figure 3:**
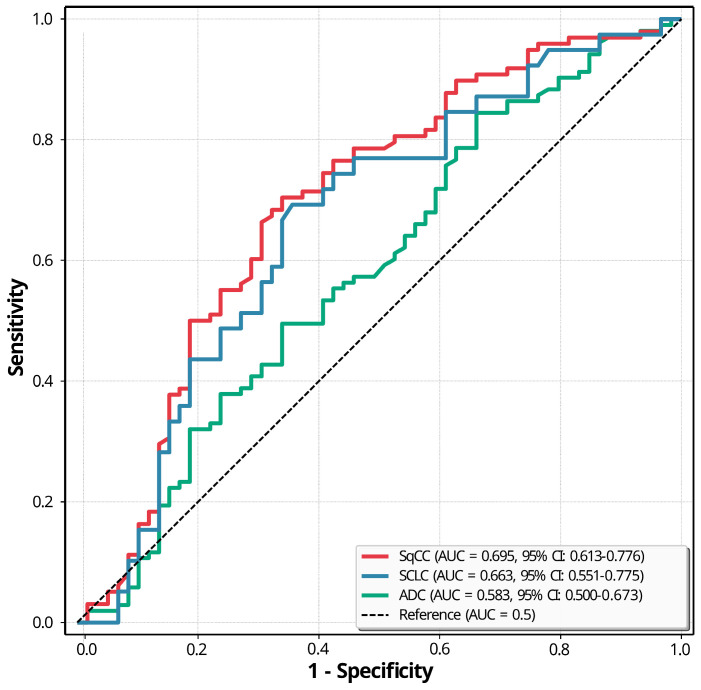
Receiver operating characteristic (ROC) curves of the AptoDetect™-Lung assay by histologic subtype in the high-risk subgroup. ROC curves show the discriminative performance of AptoDetect™-Lung for detecting each histologic subtype versus benign lesions among participants in the high-risk Korean subgroup. The assay demonstrated higher discriminative performance for squamous cell carcinoma (SqCC; AUC 0.695; 95% CI, 0.613–0.776) and small-cell lung cancer (SCLC; AUC 0.663; 95% CI, 0.551–0.775) than for adenocarcinoma (ADC; AUC 0.583; 95% CI, 0.500–0.673). The diagonal reference line represents chance-level discrimination (AUC = 0.5). ADC, adenocarcinoma; AUC, area under the curve; CI, confidence interval; ROC, receiver operating characteristic; SCLC, small-cell lung cancer; SqCC, squamous cell carcinoma.

Histology-specific analyses demonstrated heterogeneity in diagnostic performance across lung cancer subtypes (Figure 3). The AptoDetect™-Lung assay showed the highest discriminative ability for squamous cell carcinoma (AUC 0.695; 95% CI, 0.613–0.776) and small-cell lung cancer (AUC 0.663; 95% CI, 0.551–0.775), whereas performance was more modest for adenocarcinoma (AUC 0.583; 95% CI, 0.500–0.673). Using the manufacturer-recommended cutoff score (≥5.0), sensitivity was highest for squamous cell carcinoma (56.1%) and small-cell lung cancer (51.3%), but lower for adenocarcinoma (38.8%), while specificity remained consistent across histologic subtypes (72.9%). This pattern is consistent with known biological and molecular differences between smoking-related and non-smoking-related lung cancers.

**Figure 4:**
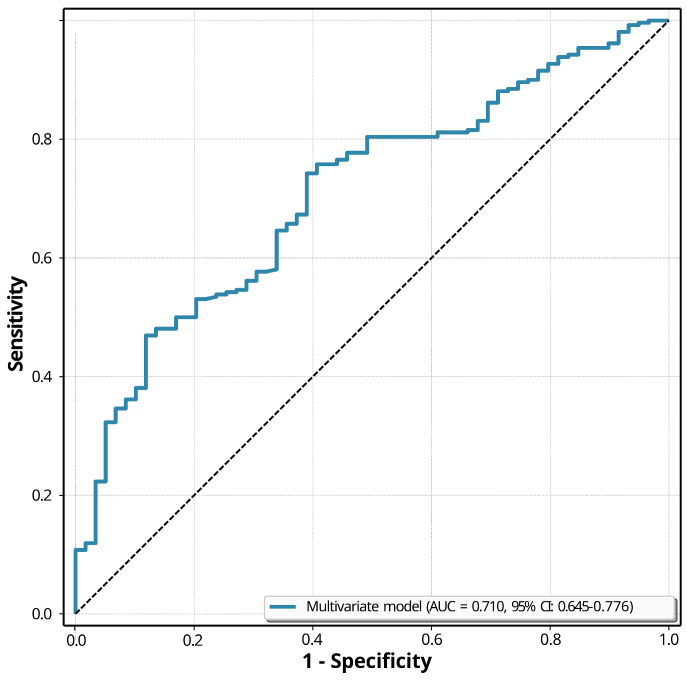
Receiver operating characteristic (ROC) curve of the multivariate model for predicting lung cancer in the high-risk subgroup. The model incorporated AptoDetect™-Lung score, Lung-RADS category (4B/4X vs. 3/4A), age, and familial cancer history. In the high-risk Korean subgroup (*n* = 319), the model achieved an AUC of 0.710 (95% CI, 0.645–0.776). The diagonal dotted reference line represents chance-level discrimination (AUC = 0.5). AUC, area under the curve; CI, confidence interval; Lung-RADS, Lung Imaging Reporting and Data System; ROC, receiver operating characteristic.

A multivariable prediction model integrating the AptoDetect™-Lung score with Lung-RADS category, age, and family history of cancer demonstrated improved discrimination in the high-risk subgroup, achieving an AUC of 0.710 (95% CI, 0.645–0.776) (Figure 4). This represents a numerical improvement over the biomarker alone and supports the potential complementary value of combining blood-based biomarkers with clinical and radiologic risk factors for lung cancer risk stratification.

## 4. Discussion

In this multicenter prospective cohort study, we evaluated the diagnostic performance of the AptoDetect™-Lung aptamer-based biomarker assay in high-risk individuals with Lung-RADS category 3 or 4 pulmonary nodules detected through LDCT screening. Although LDCT screening has been shown to reduce lung cancer-specific mortality by approximately 20% in high-risk populations [1], the high prevalence of indeterminate and false-positive findings remains a substantial clinical challenge [1,17,18]. The present study enrolled individuals who met contemporary screening eligibility criteria for high-risk populations [6], thereby enhancing the clinical relevance and translational applicability of our findings. Notably, the AptoDetect™-Lung score remained independently associated with lung cancer after adjustment for established clinical and radiologic risk factors, supporting its modest utility as a complementary tool to imaging-based risk assessment rather than a standalone diagnostic test.

To assess the generalizability of our findings and to examine whether the high-risk screening cohort represented a systematically selected subset, we compared baseline characteristics and biomarker performance between the high-risk subgroup and the overall study population. Distributions of Lung-RADS categories, aptamer scores, and the proportion of individuals classified as high risk by the assay were broadly similar between groups. These findings suggest that, with respect to Lung-RADS distribution and biomarker profiles, the high-risk subgroup was broadly comparable to the overall analytic cohort, and that the observed associations were unlikely to be explained solely by subgroup enrichment.

As anticipated, the high-risk group—defined by greater cumulative smoking exposure—demonstrated a higher prevalence of smoking-related histologic subtypes, including squamous cell carcinoma and small-cell lung cancer [19]. Notably, this study included a substantial number of patients with small-cell lung cancer, a histologic subtype that has been underrepresented in prior biomarker studies conducted on screening-detected pulmonary nodules. In histology-specific analyses, we observed variability in diagnostic performance across lung cancer subtypes, with lower discriminative ability for adenocarcinoma compared with other subtypes. This heterogeneity likely reflects underlying biologic differences among lung cancer subtypes [20]. Adenocarcinoma, particularly in early stages, often presents as small, peripheral lesions with predominantly lepidic growth patterns [21] and limited stromal invasion, which may result in weaker systemic inflammatory or proteomic signals detectable in peripheral blood. In contrast, smoking-related tumors such as squamous cell carcinoma and small-cell lung cancer are more frequently characterized by extensive tissue remodeling, necrosis, and inflammatory activation [21,22,23], potentially leading to more readily detectable circulating protein signatures. This pattern may be biologically plausible given that the AptoDetect™-Lung panel captures multiple tumor–host response pathways, including complement activation, systemic inflammation, and extracellular matrix remodeling, which may be more pronounced in smoking-related histologies than in adenocarcinoma [19]. However, this explanation remains hypothesis-generating, as the present study was not designed to determine the causal biological mechanisms underlying histology-specific performance differences and the number of cases within individual histologic subgroups was limited.

In univariate analyses, Lung-RADS category, aptamer score, and age were significantly associated with lung cancer diagnosis, while family history of cancer showed a borderline association and was therefore included in multivariable modeling. In multivariable analyses, Lung-RADS category, aptamer score, age, and family history of cancer each remained independently associated with malignancy. While the central role of radiologic assessment in lung cancer screening is well established, the persistence of the aptamer score as an independent predictor suggests that it captures biologic information not fully reflected by imaging features alone. Notably, family history of cancer emerged as an independent risk factor even within a population of heavy smokers. Previous studies have largely emphasized susceptibility in never-smokers [24,25], in whom genetic predisposition is thought to play a more prominent etiologic role. Our findings extend this concept by suggesting that inherited risk may also contribute to lung cancer development among smokers, despite substantial tobacco exposure. This observation underscores the multifactorial nature of lung cancer risk and supports the incorporation of family history into comprehensive risk assessment models, even in smoking-enriched screening populations [26,27].

Although the discriminative performance of the AptoDetect™-Lung assay alone was modest, with an AUC of 0.570 in the total cohort and 0.639 in the high-risk subgroup, the biomarker score remained independently associated with malignancy after adjustment for Lung-RADS category, age, and family history. Moreover, when incorporated into a multivariable model with clinical and radiologic variables, the combined model achieved an AUC of 0.710, supporting the potential value of a multimodal risk assessment strategy. These findings suggest that AptoDetect™-Lung may capture serum protein-based biological information that is not fully reflected by conventional clinical and imaging-based risk factors, supporting its potential role as one component of integrated pulmonary nodule risk assessment.

The internal validation results provide cautious support for the reliability of the multivariable model within the study population. Bootstrap resampling with 1000 iterations confirmed the stability of the apparent AUC. The calibration intercept (0.007) and slope (0.991) were close to their ideal values, suggesting reasonable agreement between predicted and observed probabilities. However, the Hosmer–Lemeshow test was statistically significant (*p* = 0.016), indicating some departure from perfect calibration. This finding should be interpreted in the context of the high malignancy prevalence and specific Lung-RADS distribution of the analytic cohort. Although the model showed acceptable internal performance, external validation in more representative screening cohorts will be necessary to assess generalizability and calibration stability across different clinical settings.

The clinical implication of these findings is not that AptoDetect™-Lung should be used as a standalone diagnostic test, but rather that blood-based biomarkers may have potential as adjunctive tools within integrated nodule evaluation. In particular, the assay may complement established approaches such as Lung-RADS-based radiologic assessment by providing additional biological information during the evaluation of LDCT-detected nodules. Although selected clinical and radiologic variables were incorporated into the multivariable model, pre-specified formal comparison with established pulmonary nodule prediction models or routinely used biomarkers was not performed. Future studies should evaluate the incremental value of AptoDetect™-Lung within existing clinical, radiologic, and biomarker-based risk stratification frameworks.

The composition of the analytic cohort should be considered when interpreting these results. Because the analysis was restricted to participants with histopathologic confirmation, patients with lower-suspicion nodules managed by imaging surveillance were underrepresented. This requirement strengthened outcome ascertainment and reduced the risk of disease misclassification, but it also enriched the study population for higher-risk nodules. The resulting malignancy prevalence of 81.5% in the high-risk subgroup and the predominance of Lung-RADS category 4B (41.1%) and 4X (49.5%) nodules, with very few category 3 nodules (1.6%), reflect this selected diagnostic setting. Accordingly, the study provides evidence supporting an association between the biomarker score and malignancy in a high-suspicion nodule cohort, but the reported diagnostic performance estimates, including sensitivity, specificity, predictive values, and AUC, should not be directly extrapolated to unselected LDCT screening populations without further validation. Prospective studies in broader intended-use cohorts, particularly those including lower-suspicion nodules managed by surveillance, will be necessary to establish the generalizability and clinical applicability of these findings.

**Figure 5:**
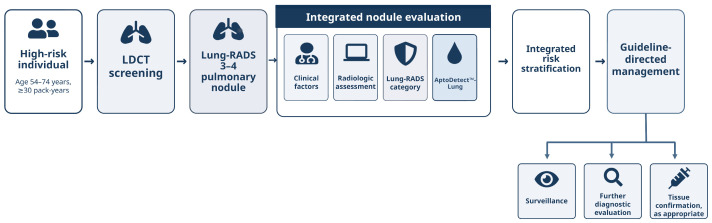
Proposed adjunctive role of AptoDetect™-Lung in the evaluation of LDCT-detected pulmonary nodules. This schematic illustrates the potential adjunctive role of AptoDetect™-Lung as part of integrated risk assessment for LDCT-detected pulmonary nodules, together with clinical factors, radiologic evaluation, and Lung-RADS categorization. Subsequent management should follow established guideline-directed clinical pathways. LDCT, low-dose computed tomography; Lung-RADS, Lung Imaging Reporting and Data System.

Based on these findings, Figure 5 presents a conceptual workflow for the adjunctive use of AptoDetect™-Lung in the evaluation of LDCT-detected pulmonary nodules. The assay is positioned alongside clinical factors, radiologic assessment, and Lung-RADS categorization as one component of integrated nodule evaluation. The downstream management options shown in the figure, including surveillance, further diagnostic evaluation, and tissue confirmation, should be interpreted within existing guideline-directed management pathways. This framework is intended to illustrate a potential role for the assay in multimodal risk stratification rather than to propose a prescriptive clinical algorithm.

Several limitations should be considered. First, although this was a multicenter prospective study, the number of lung cancer events was limited, constraining statistical power for detailed subgroup analyses, particularly by histologic subtype. Second, detailed information on concomitant medications and comorbid conditions was not systematically collected, and their potential influence on biomarker measurements could not be fully excluded. Third, independent external validation was not performed. Therefore, the performance of the AptoDetect™-Lung assay and the integrated prediction model may not generalize across different populations, screening settings, or healthcare systems. Fourth, direct comparisons with established pulmonary nodule risk prediction models or other validated blood-based biomarkers were not performed, leaving the incremental value of AptoDetect™-Lung relative to current standards to be determined. Additionally, the predominance of Lung-RADS 4B/4X nodules limited evaluation of biomarker performance in lower-suspicion nodules. Further validation in more representative LDCT screening cohorts will be required to clarify its role in screening workflows.

## 5. Conclusions

In conclusion, this multicenter prospective study showed that AptoDetect™-Lung was independently associated with malignancy in high-risk individuals with LDCT-detected pulmonary nodules after adjustment for imaging and clinical risk factors. Despite its modest standalone discriminative performance, the assay may have value as an adjunctive risk-stratification tool. Further validation in broader intended-use populations is needed before clinical implementation can be considered.

## Figures and Tables

**Table 1:** Baseline characteristics of participants in the total cohort and the high-risk subgroup.

Variables	Total (*n* = 1084)	High-Risk Patients (*n* = 319)	*p*-Value
Age, years	69.0 (62.0–75.0)	66.0 (62.0–70.5)	<0.001
Male sex	738 (68.1%)	309 (96.9%)	<0.001
ECOG (0–1)	1055 (97.3%)	315 (98.7%)	0.20
BMI (kg/m^2^)	23.84 (±3.45)	24.02 (±3.37)	0.40
Smoking history	1083	319	<0.001
Never	381 (35.2%)	0 (0.0%)	
Former	315 (29.1%)	99 (31.0%)	
Current	387 (35.7%)	220 (69.0%)	
Pack-years	37.5 (20.0–46.0)	42.0 (38.0–50.0)	<0.001
Cancer history	58 (5.4%)	21 (6.6%)	0.41
Familial cancer history	355 (33.1%)	109 (34.2%)	0.68
Pulmonary history	195 (18.0%)	67 (21.0%)	0.26
Lung-RADS	1083	319	0.20
3	19 (1.8%)	5 (1.6%)	
4A	94 (8.7%)	25 (7.8%)	
4B	507 (46.8%)	131 (41.1%)	
4X	463 (42.8%)	158 (49.5%)	
Aptamer score	4.2 (1.9–7.2)	4.4 (2.2–7.5)	0.37
Aptamer score ≥ 5	457 (42.2%)	140 (43.9%)	0.63
CEA (ng/mL)	3.4 (2.0–7.4)	3.9 (2.3–7.9)	0.05
Histology	868	260	<0.001
ADC	535 (61.6%)	103 (39.6%)	
SqCC	194 (22.4%)	98 (37.7%)	
SCLC	87 (10.0%)	39 (15.0%)	
Other	52 (6.0%)	20 (7.7%)	

High-risk subgroup was defined based on the Korean low-dose computed tomography screening criteria: age 54–74 years with ≥30 pack-years of smoking history. Values are presented as median (interquartile range), mean ± standard deviation, or n (%) as appropriate. *p*-values were calculated using the Mann–Whitney U test for continuous variables and chi-square test for categorical variables (Fisher’s exact test when expected cell count < 5). AptoDetect™-Lung score represents the quantitative biomarker output; scores ≥ 5 are categorized as high-risk for descriptive purposes based on manufacturer recommendations. Smoking history: never-smoker (no lifetime tobacco exposure); former smoker (quit ≥ 1 year before enrollment); current smoker (active smoking or quit within <1 year). Cancer history refers to a personal history of any malignancy other than lung cancer. Familial cancer history denotes cancer in first-degree relatives (parents, siblings, or children). Pulmonary history includes non-malignant chronic lung diseases such as tuberculosis, chronic obstructive pulmonary disease, or interstitial lung disease. Abbreviations: ADC, adenocarcinoma; BMI, body mass index; CEA, carcinoembryonic antigen; ECOG, Eastern Cooperative Oncology Group performance status; Lung-RADS, Lung Imaging Reporting and Data System; SCLC, small-cell lung cancer; SqCC, squamous cell carcinoma.

**Table 2:** Univariate analysis of predictors of lung cancer in the high-risk subgroup.

Variables	OR (95% CI)	*p*-Value
AptoDetect™-Lung assay score	1.190 (1.070–1.323)	0.001
High-risk Lung-RADS category (4B/4X vs. 3/4A)	2.907 (1.300–6.499)	0.009
Age (per year)	1.081 (1.027–1.139)	0.003
Familial cancer history	1.851 (0.965–3.548)	0.064
Personal cancer history	0.708 (0.249–2.017)	0.518
ECOG ≥ 2	0.677 (0.069–6.626)	0.738

Each variable was analyzed separately using univariate logistic regression. Variables with *p* < 0.10 were considered for inclusion in the multivariate model (Table 3). OR, odds ratio; CI, confidence interval.

**Table 3:** Multivariate logistic regression analysis of predictors of lung cancer in the high-risk subgroup.

Variables	OR (95% CI)	*p*-Value
AptoDetect™-Lung assay score	1.139 (1.020–1.272)	0.020
High-risk Lung-RADS category (4B/4X vs. 3/4A)	2.726 (1.158–6.416)	0.022
Age (per year)	1.084 (1.025–1.145)	0.005
Familial cancer history	2.171 (1.093–4.313)	0.027

Variables with *p* < 0.10 in univariate analysis (Table 2) were included in the multivariate model. Adjusted odds ratios (OR) with 95% confidence intervals (CI) are reported. Model performance: AIC = 288.3, McFadden R^2^ = 0.089, likelihood ratio χ^2^ = 27.2 (*p* < 0.001).

## Data Availability

The data presented in this study are available on request from the corresponding author. The data are not publicly available due to privacy restrictions.

## Abbreviations

The following abbreviations are used in this manuscript:

BMI	Body mass index
CA6	Carbonic anhydrase VI
CEA	Carcinoembryonic antigen
CI	Confidence interval
CRP	C-reactive protein
C9	Complement component 9
ECOG	Eastern Cooperative Oncology Group
EGFR1	Epidermal growth factor receptor 1
IQR	Interquartile range
KIT	KIT proto-oncogene, receptor tyrosine kinase
LDCT	Low-dose computed tomography
Lung-RADS	Lung Imaging Reporting and Data System
MMP7	Matrix metalloproteinase 7
NPV	Negative predictive value
NTM	Nontuberculous mycobacteria
PPV	Positive predictive value
SD	Standard deviation
SERPINA3	Serpin family A member 3 (α1-antichymotrypsin)
